# A Role for RNA Viruses in the Pathogenesis of Burkitt's Lymphoma: The Need for Reappraisal

**DOI:** 10.1155/2012/494758

**Published:** 2011-11-29

**Authors:** Corry van den Bosch

**Affiliations:** Research Facilitation Forum, Pilgrims Hospices, Canterbury, Kent CT2 8JA, UK

## Abstract

Certain infectious agents are associated with lymphomas, but the strength of the association varies geographically, suggesting that local environmental factors make important contributions to lymphomagenesis. Endemic Burkitt's Lymphoma has well-defined environmental requirements making it particularly suitable for research into local environmental factors. The Epstein-Barr virus and holoendemic Malaria are recognized as important cofactors in endemic Burkitt's Lymphoma and their contributions are discussed. Additionally, infection with Chikungunya Fever, a potentially oncogenic arbovirus, was associated with the onset of endemic Burkitt's Lymphoma in one study and also with space-time case clusters of the lymphoma. Chikungunya Virus has several characteristics typical of oncogenic viruses. The Flavivirus, Hepatitis C, a Class 1 Human Carcinogen, closely related to the arboviruses, Yellow Fever, and Dengue, is also more distantly related to Chikungunya Virus. The mechanisms of oncogenesis believed to operate in Hepatitis C lymphomagenesis are discussed, as is their potential applicability to Chikungunya Virus.

## 1. Introduction

It has been estimated that approximately 20% of all cancers, worldwide, are attributable to infectious agents [1]. This is likely to be an underestimate because of under-reporting and under-ascertainment, particularly in resource-poor countries, where the burden of infection-related cancers is almost four times that of the more prosperous countries [1]. A number of infectious agents, comprised of a variety of different types of organisms, have been shown to be associated with lymphomas. It is highly probable that this number will continue to expand as diagnostic methods improve, new organisms emerge and general advances in knowledge are made. 

Some of the organisms which have been linked with different types of lymphomas have already been designated Class 1 Human Carcinogens by the World Health Organisation. They are the DNA Herpes viruses, the *Epstein-Barr virus* [2] and *Kaposi Sarcoma Herpesvirus* [2, 3], the retroviruses *Human Immunodeficiency Virus type 1* and *Human T Cell Lymphotropic Virus Type 1 *[4], the Hepatitis viruses, *Hepatitis B*, a DNA virus, and *Hepatitis C*, an RNA virus [5], and the bacterium, *Helicobacter pylori* [6]. In addition, the bacteria *Campylobacter jejuni* [7], *Chlamydia psittaci* and *pneumoniae* [8, 9], *Borrelia burgdorferi* [10, 11] and the RNA Alphavirus *Chikungunya virus* [12], an arbovirus, have been found to be associated with various different forms of lymphoma. The *Epstein-Barr virus* (EBV) [2], the protozoon, *Malaria* [13], and the vector-borne Alphavirus, *Chikungunya virus* (CHIKV), have been linked specifically with endemic Burkitt's Lymphoma (eBL), perhaps the best studied of all lymphomas. Studies of associations between lymphomas and different infectious organisms often show considerable geographic differences in the strength of the association, suggesting that local environmental factors, including lifestyle-related ones, as yet unidentified, may play important roles in lymphomagenesis [9, 14, 15].

The infectious agents linked with lymphomas are thought to promote lymphomagenesis by processes linked with chronic antigenic stimulation. They establish persistent infections, accompanied by overt or silent chronic inflammation, leading to cytokine activity, the activation of cyto-oncogenes, with or without chromosomal abnormalities, and the inactivation of tumour-suppressor genes [16–18]. Some viruses, including EBV and *Hepatitis C* (HCV) [19], can cause a polyclonal B cell proliferation, a risk factor for Non-Hodgkins Lymphomas. Immunosuppression may be important, as with HIV-associated Lymphomas [18]. Oncogenic viruses may or may not appear to co-operate: in HIV infection, the incidence of EBV-positive Burkitt's Lymphoma is increased [18], whereas that of HCV-associated lymphomas is reduced [19, 20]. 

As we learn more, our understanding of the process of oncogenesis is changing from the view that it is confined to a series of irreversible genetic changes in the cell, culminating in full-blown malignancy, to an appreciation of the important contribution made by epigenetic changes and the balance of forces promoting or opposing apoptosis, many driven by infectious agents. Some of these changes are reversible, and, in a few cases, and under certain conditions, the process of oncogenesis can be reversed, as will be discussed later. 

This paper will concentrate on those aspects of lymphomagenesis, particularly apparent co-operation between cofactors, which are best exemplified in endemic Burkitt's Lymphoma (eBL), often described as the “Rosetta Stone” of cancer [21]. It will discuss, drawing on research into lymphomagenesis in HCV infection, how the arbovirus, CHIKV, shown to be associated with the onset of eBL [12, 22], might contribute to lymphomagenesis.

## 2. Burkitt's Lymphoma

Burkitt's Lymphoma (BL), an aggressive non-Hodgkins Lymphoma (NHL), has an extremely rapid doubling time of 24–48 hours as almost all the cells are cycling at one time [23]. It has been calculated, based on the phenomena of seasonality and time-space case clusters sometimes observed in the endemic form of Burkitt's Lymphoma (eBL), that the latent period for this lymphoma is likely to be as short as one year [24]. The rapid growth, coupled with a short induction period could, theoretically, make the train of events involved in lymphomagenesis easier to unravel. 

There are three types of BL: endemic or “African,” sporadic and HIV-associated. BL can also arise in association with severe immunosuppression as in organ transplants [25, 26]. All BLs have one of three translocations, of which *t*(*8:14*) is by far the commonest. They involve the *C-MYC* oncogene on the long arm of chromosome 8, and an immunoglobulin chain gene [27]. The *C-MYC* gene, which plays an important role in cellular proliferation, becomes deregulated and activated as a consequence of the BL translocation. This occurs due to proximity to Ig transcriptional enhancers [28] or to structural alterations within *C-MYC* [29]. The linking of *C-MYC* to immunoglobulin sequences leads to constitutive *MYC* expression and the cell is unable to leave the cycling phase [30]. Although a deregulated *C-MYC* plays an important role in lymphomagenesis in eBL [31], working in conjunction with the EBV [32], it cannot institute tumorigenesis unaided [33]. The *TP53* mutations, commonly found in BL, may be accompanied with a gain in transforming ability and loss of growth suppression [34, 35], but are thought to contribute to tumour progression rather than lymphomagenesis.

Endemic and sporadic forms of BL have different breakpoints within both the *MYC* locus on chromosome 8 and the Ig heavy-chain locus on chromosome 14. There are also clinical, molecular, and cytological differences and varying degrees of EBV positivity, which exhibit a geographic gradient [14]. These differences, together with the well-defined climatic requirements for the endemic form, highlight the probable importance of as yet unrecognised lymphomagenic environmental factors, which may differ throughout the world. 

BL cells are B lymphocytes with rearranged immunoglobulin genes, secreting immunoglobulin chains which correlate with the site involved in the translocation, suggesting that an active immunoglobulin locus is directly involved [36]. Cloning of translocation breakpoints from endemic cases has revealed evidence of V-D-J (variable diversity joining) recombinase involvement in the genesis of the translocations [37] strongly suggesting that eBL arises while the cell is actively arranging its IgG genes. It appears that the deletions and insertions seen in Ig V_H_DJ_H_ mutations occur as the result of an antigen-driven selection process and that the *C-MYC*/Ig translocation happens due to hypermutation in B-cells entering or transiting Germinal Centres [35, 38, 39].

### 2.1. Lymphomagenesis of Endemic Burkitt's Lymphoma

It is thought that lymphomagenesis in eBL begins in infancy. Heavy primary EBV infection results in a degree of immune tolerance. EBV-infected cells proliferate and some are immortalised and transformed [40]. Heavy malarial infection further stimulates expansion of the B-cell pool and suppresses T cells involved in EBV control. The final stage of lymphomagenesis is the development of the characteristic translocation, leading to deregulation of *C-MYC* and the development of a malignant clone.

#### 2.1.1. EBV and Lymphomagenesis

Much has been learned about the mode of action of EBV in oncogenesis, but much remains to be learned [41]. EBV is associated with various forms of Non-Hodgkins Lymphoma (NHL), including Post-transplant lymphomas, some AIDS-related large-cell lymphomas, BL, and also Hodgkins Lymphoma. The extent of the association varies geographically by type of lymphoma and location. The evidence for a causal relationship is the strongest with eBL where the EBV genome is incorporated into 90% or more of cases in the African Lymphoma Belt [42]. No preferential integration site in the human chromosome [43] has been shown, the virus being integrated into the genome at a number of different sites in cell lines, but human-mouse hybrid cell studies suggest that the EBV genome is consistently associated with Chromosome 14 [44].

#### 2.1.2. EBV-Antibody Studies

EBV seems to be actively involved in all stages of eBL development, as judged by EBV-antibody responses. The association between eBL and raised levels of EBV-VCA (Viral Capsid Antigen) and EBV-EA (Early Antigen) antibodies, both associated with actively replicating virus, is one factor implicating EBV as an active participant in lymphomagenesis. In a Ugandan prospective study, children who subsequently developed BL had significantly higher titres of EBV-VCA IgG antibodies up to 6 years before the onset of the lymphoma [45, 46], and these were the only antibodies showing a significant increase. There was no further rise after the onset of BL. Chronic rather than acute EBV infection appeared to be relevant to lymphomagenesis. EBV-EA antibody levels were shown to increase as the tumour grew, and to decline after treatment [47], again implicating an active rather than a latent phase of EBV infection. Additionally, EBV-specific antibody-mediated cellular cytotoxicity appeared to have a prognostic significance for BL patients [48]. 

Raised levels of antibodies to EBV-VCA and EBV-EA antigens were also found in the relatives of BL patients [49], those exposed to chronic malaria [50, 51] and users of herbal remedies [52], which included plants producing tumour-promoter substances with EBV-potentiating activity. *Euphorbia tirucalli* is one of a number of such plants growing commonly in Africa and, more notably, around the homes of BL patients [53, 54]. It is possible that exposure to these plants, which secrete their active principles into the soil [55, 56], thus potentially contaminating environmental air and water, could account for some of the rises in EBV-antibodies seen in eBL. It has been suggested that the relatives of BL patients have raised EBV-antibody titres [49] because they share a similar immune dysfunction, due to similar exposures to environmental factors.

#### 2.1.3. Potential Contributions of EBV to Lymphomagenesis

The EBV could potentiate lymphomagenesis in several ways. EBV is able to stimulate and maintain B-cell proliferation because of CD40 and B Cell Receptor (BCR) mimicry, increasing the size of the B cell pool, and, thereby, the chances of translocations and other cytogenetic changes occurring [57]. EBV can immortalize and transform lymphocytes and may also collaborate, in as yet unidentified ways, with the changes induced by the *C-MYC* translocation [32, 58]. EBV proteins such as EBNA1 may induce epigenetic changes, with subsequent cellular dysregulation [59, 60]. EBV also encodes products which can interact with, or mimic, a variety of cellular molecules, signals, and cytokines, many of which have antiapoptotic actions, and which promote lymphomagenesis [18, 30, 57, 61–63]. EBV infection also protects cells damaged by mutations from destruction by apoptosis, thus allowing them to replicate [62] and this function may be extremely important in lymphomagenesis [63, 64].

#### 2.1.4. EBV Infection and Immunological Control

Over 90% of the world's population is infected with EBV. Once infected, people become lifelong carriers of the virus which persists in two main forms: circulating latently infected cells and a localized lytic infection in epithelial cells in the mouth and pharynx, possibly also the urogenital tract and salivary glands [65]. In generalized immunodeficiency states such as HIV infection and transplant patients, or the more specifically EBV-linked Duncan's Syndrome, proliferation of B-cells can proceed unchecked [42, 66] and may evolve from a polyclonal reactive process to a monoclonal malignant lymphoma [67]. The polyclonal activation and proliferation subsequent upon primary EBV infection is normally controlled by inhibitory immunological mechanisms, as it is usually followed by the development of cellular immunity and antibodies to the various EBV antigens. Killer cells and EBV-specific cytotoxic lymphocytes are also generated, the latter playing a crucial role in controlling circulating EBV-infected cells [68]. It has also been suggested that, because BL cells have a “resting” rather than B-blast phenotype, together with the accompanying changes in expression of certain EBV, HLA and adhesion molecules, the BL cell is not rejected by the EBV-specific immune response [30].

#### 2.1.5. EBV Latency

After the acute infection has subsided, a type of EBV latency is found where most latency transcripts are undetectable [65]. However, BL cell lines display a unique Type I latency where the EBV nuclear protein, EBNA1, and the EBV RNA transcripts, the EBERs, and BART (BamA rightward transcripts) are expressed [69, 70]. However, some authorities believe that the concept of BL cells predominantly exhibiting type I latency is an oversimplification [71–73].

EBNA1 is responsible for maintaining the EBV episome in latently infected cells [74]. There are EBNA1 binding sites in the human genome and, as EBNA1 can bind both RNA and DNA, it could influence the expression of viral or cellular genes [75], possibly by eliciting demethylation and subsequent activation or dysfunction of cellular functions [59]. EBNA1 can up regulate the recombinase-activating genes which mediate V-D-J combination and are usually only expressed in immature lymphoid cells [76]. EBNA1 is indispensable for B cell transformation and can enhance B cell immortalization several thousandfold [77]. Although EBNA1 does not appear to be oncogenic on its own, as it is consistently expressed in EBV-infected cells, including latently infected cells, without oncogenic sequelae, EBNA1 transgenic mice can develop monoclonal B-cell lymphomas similar to those induced by transgenic *C-MYC* expression [78]. EBNA 1 and *MYC*, the murine analogue of the human oncogene *C-MYC*, seemed to cooperate in lymphomagenesis in a transgenic mouse model, suggesting the possibility of a similar action in man [32]. 

The RNA transcripts, EBERs 1 and 2, appear to produce resistance to apoptosis, conferring a malignant phenotype [79, 80]. They can modulate expression of LMP1 considered to be the EBV oncogene [81], upregulate *BCL-2*, inhibit apoptosis by binding protein kinase, block apoptosis due to Interferon-*α* signalling, stimulate production of Interleukin (IL)-10, an autocrine growth factor for BL cells, induce colony growth of cells in agar, and are tumorigenic in immunodeficient mice [58, 82, 83]. In addition, a binding site for *C-MYC*, found in the promoter for EBER 1, permits cooperation with *C-MYC* and a role in lymphomagenesis [58]. LMP1, only found in a small minority of BLs, but uniformly present in Naso-Pharyngeal Carcinoma, has transforming ability, is tumorigenic in nude mice [84], inhibits apoptosis in B lymphocytes, and induces expression of the antiapoptotic *BCL2* oncogene [85].

### 2.2. Role of Malaria

In sub-Saharan Africa, 90% of children are infected with EBV by the age of 2 years and have a degree of immune tolerance to it [86] which is exacerbated by the immunomodulatory effects of chronic malaria. Holoendemic malaria undoubtedly contributes to the greatly increased numbers of BL cases, nearly all EBV-positive, seen in the Lymphoma Belt. Malaria produces polyclonal B cell activation [87], a five-fold increase in EBV-positive cells during acute malarial infection [88], inhibition of EBV-specific cytotoxic T cells [89], an increase in EBV-transformed B cells [89], and higher circulating levels of EBV-positive cells in children [51].The combination of EBV and holoendemic malaria has been credited with amplifying the incidence of BL in African children approximately a hundred-fold. Rates of BL are 0.04–0.08/100,000 in Western Europe, increasing to 1-2/100,000 in countries of intermediate prevalence such as Algeria, and up to 10/100,000 in the African Lymphoma Belt [86]. Similarly, BL EBV-positivity ranges from 10–15% in France, up to 85% in Algeria and over 90% in the Lymphoma Belt of Africa [86].

### 2.3. Arboviruses and the Epidemiology of Endemic Burkitt's Lymphoma

While it is recognized that EBV and malaria make important contributions to BL endemicity, yet the sporadic form of BL can occur in the absence of both of these infections and, if early EBV infection and Holoendemic malaria are the only prerequisites for eBL, then the tumour should be much commoner than it is within the African Lymphoma Belt, where malarial transmission is intense. The Belt lies between the latitudes 10° north and south of the equator with an extension along the eastern coastal margin of Mozambique. BL is endemic within the Lymphoma Belt wherever mean minimum temperatures exceed 15.5°C and annual rainfall is above 50 mls [90, 91]. The lymphoma appears to be associated with water and is absent from arid areas [92]. The climatically defined Lymphoma Belt coincides with the geographic distribution of holoendemic malaria, vectors of certain arboviruses such as Chikungunya Virus (CHIKV) [93], and EBV-activating plants such as *Euphorbia tirucalli* [54], all of which conform with one of Chapin's zones of flora and fauna [90, 91, 94].

Endemic BL exhibits unusual features such as seasonality [95, 96], shifting foci, or lymphoma “hot-spots” which change location from year to year [97, 98] and both spatial [99, 100] and space-time case clusters [95, 97, 98, 101–104]. The clustering was very striking when it occured; in the Aliba outbreak, four of the five cases from this small village presented within one year [101] and unrelated cases in clusters in Malawi were often very close in space and time, with one unrelated case-pair living in neighbouring huts. Statistically significant clustering at intervals of less than 2.5 kms and less than 60 days was seen in Malawi [105]. Clustering was more pronounced in older children in both Uganda and Malawi [98, 104]. The case clusters are summarized in Table 1.

Although the phenomena of seasonality, shifting foci, and clustering have been observed and are well documented, they are not always found, even when sought [103, 105]. Clustering can best be explained by an environmental cofactor which moves around and is variable from year to year, such as an infectious disease, especially one like measles which can cause epidemics and clusters [101]. Neither malaria, nor infections with other common parasites such as Schistosoma, a Class 1 human carcinogen, can provide a convincing explanation for the phenomena. Heavy Schistosomal infection exerts a considerable effect upon the immune system and could potentially contribute to lymphomagenesis by inducing a skewing of the immune response away from the TH1 cell-mediated immunosurveillance towards a B-lymphocyte dominated TH2 response [106]; indeed Schistosomal lesions adjacent to BL lesions have been noted [107]. Foci of Schistosomal infections could explain spatial clustering, but not space-time clustering or shifting foci of BL cases. However, insect-borne viruses, known as arboviruses, are particularly well suited to explain the occurrence of space-time case clusters. The epidemiology of eBL mimics that of certain arboviruses, including their temperature requirements, age, and geographic distributions more closely than that of malaria [22, 91, 108], as shown in Table 2. An arbovirus, which is endemic, but causes periodic epidemics, could explain the existence of the time-space case clusters during an epidemic, and their absence, in the intervening periods, when it is endemic [22, 56]. Morrow et al. [105] observed that the incidence of the tumour was inversely related to age, suggesting that intense malarial transmission was associated with earlier age of onset. This observation could also apply to arboviral infection as both infections are increased where mosquitoes thrive. Both malaria and most arboviruses are vectored by mosquitoes. 

### 2.4. Space-Time Case Clusters of Endemic Burkitt's Lymphoma

A statistically significant association between infection with the arbovirus, CHIKV and the onset of eBL was observed in Malaŵi, at the time of a CHIKV epidemic, when space-time case clusters were also being observed [12]. BL patients were significantly more likely to be CHIKV seropositive on first admission or to have seroconverted three weeks afterwards than either hospital or local controls (*P* = 0.002 and 0.009, resp.) [12]. A majority of BL patients, irrespective of CHIKV seropositivity, gave a history of signs and symptoms typical of arboviral infection, such as rashes, oral lesions, and bleeding tendencies, occurring shortly before the appearance of BL, as summarized in Table 3 [12, 22, 56]. Rashes and oral lesions preceding BL onset had been seen previously in Uganda and attributed to Herpes or Measles infection [110]. However, most unimmunized children in tropical Africa acquire measles by 1–4 years, whereas BL is not seen before the age of 2-3 years, peaks at 5–8 years, depending on the degree of endemicity and is rarely seen after the age of 18 years [110].

Ugandan BL serological studies showed that antibodies to various arboviruses, which included CHIKV, were significantly more likely to be found in BL patients, and to a lesser extent, their families, than controls, but no one arbovirus predominated [109, 111]. This would be consistent with more than one arbovirus being associated with BL. This possibility is also suggested by the observation that three patients, seronegative for CHIKV, seroconverted for Yellow Fever during the course of their first admission, and other cases, seronegative for both viruses, had high titres of antibody to Sandfly Fever, denoting recent infection [56]. Additionally, some Ugandan space-time case clusters [101] occurred during or following an epidemic of O'nyong-nyong, an arbovirus closely related to CHIKV [112, 113] and others [95, 97, 98, 101–103] also occurred during periods when CHIKV activity was recorded in East and Central Africa, viz. 1958, 1960-61, 1963-65, 1967, 1971, 1973 [114].

## 3. Arboviruses

Arboviruses occur world-wide, particularly in the tropics and where vector-control is poor. They are an important group of diseases, with considerable economic consequences for the livestock industry [115], and a considerable burden of morbidity and mortality in humans [115, 116] although human disease is too often unrecognized or misdiagnosed [116, 117], except when large-scale epidemics occur as with the frequent outbreaks of Dengue in South-East Asia [118], or the recent CHIKV epidemic in the Indian Ocean [93]. Climatic conditions are important in determining arboviral outbreaks, with rainfall pattern, temperature, and humidity all playing a role [118].

There are many arboviruses, but only a minority are of medical importance. Arboviruses are RNA viruses, dependent on arthropod hosts for their transmission. They are classified on the basis of antigenic relationships, structure and manner of replication, into five main groups shown in Table 4 [119]. There is considerable cross-reactivity among different, but related, arboviruses. Viral reassortment is thought to occur in nature and, possibly, to explain the origin of some of these viruses [120].

### 3.1. Characteristics of Arboviral Infection

Arboviruses are best known for causing acute febile illnesses and only recently has the magnitude of long-term arthritic, ocular, and central nervous system sequelae, as seen in the recent Indian Ocean CHIKV epidemic [121–123], been fully appreciated [124, 125]. Subclinical infection occurs frequently and persistent infection is extremely common [121, 126, 127]. Disease is most severe in the very young and the elderly. Arboviruses can produce immunosuppression which is dependent on the age of the patient and the degree of leukopenia induced by the virus [128]. In the presence of mosquito saliva, the natural route of infection, CHIKV can skew the immune response towards the TH2 type postulated to be a risk factor for BL [106, 129]. Arboviruses can also produce the phenomenon of immune enhancement whereby pre-existing, nonneutralising, viral antibodies, due to prior infection with a different, but related serotype, enhance viral replication [130, 131], facilitating viral entry into cells and promoting the release of cytokines [132], thus increasing severity of disease. Certain strains and genotypes may be more virulent, or replicate at a higher rate, and thereby exacerbate disease severity [133]. Both the Flavivirus Dengue, and the Alphavirus CHIKV, can cause a severe form of the disease known as “Shock Syndrome” [130, 131]. Dengue serotype-crossreactive CTL clones showing high avidity for antigen produce higher levels of inflammatory cytokines than serotype-specific clones [133]. *In vitro* experiments show that Alphavirus infection inhibits host protein synthesis drastically, whilst virally encoded genes are expressed liberally [134]. Alphaviruses, Flaviviruses, and Reoviruses are particularly well suited to be vectors for heterologous genes. They are being investigated as vectors for miscellaneous treatments and vaccines and show considerable promise. However, caution needs to be exercised in view of their propensity for mutation, reassortment and establishing persistent infections [134–136].

### 3.2. Oncogenic Potential of Arboviruses

Arboviruses have the potential to be oncogenic since they exhibit persistence *in vivo* and [121] and *in vitro* [125, 137]. Persistence is enhanced, *in vitro*, if arboviruses are cultured in EBV-infected cell lines as EBV opposes the arboviral tendency to apoptosis [138]. Mice brain cells infected with CHIKV showed loss of contact inhibition and morphological alterations suggesting they had been transformed [137]. Viral isolates related to CHIKV and Bunyamwera induced tumours when injected into Swiss albino mice which could be transmitted to other animals [139]. In a series of early experiments inspired by the arboviral cofactor hypothesis, Reoviruses, which are classified as arboviruses [119], were detected in ten BL biopsies. Antibodies to Reovirus type 3 were commoner in BL cases than in controls [140–142], but no clear-cut relationship between high levels of Reovirus childhood infection and BL incidence could be established [143]. Reoviruses were reported as inducing a lymphoma in a rabbit [144, 145] and BL-like lesions in mice [146–148], but it was finally decided the tumours were induced by a Murine Leukaemia virus, the Reoviruses having been commensals [149]. 

Acute infection with the arbovirus, West Nile virus, can potentiate the actions of the tumour promoter, TPA, 12-o-tetradecanoylphorbol-13-acetate, when applied to the skin of nude mice, producing an increase in the number and size of papillomata [150]. TPA is derived from a Euphorbia, one of the EBV-activating plants considered potential cofactors in eBL lymphomagenesis [22, 151, 152].

## 4. Hepatitis C

It has already been mentioned that the Flavivirus, HCV, a Class 1 Human Carcinogen [5] is most closely related not only to Hepatitis G, another apparently oncogenic Flavivirus, but also to the Arboviruses, Yellow Fever, and Dengue [153–156]. Hepatitis G accounts for up to 9% of all NHLs in some studies, showing a stronger association with lymphomas than HCV in several studies [157, 158]. 

HCV, a Hepacivirus [155], belonging to the Flaviviridae family, produces a chronic infection, often relatively silent in the majority of cases, which persists despite the production of antibody. Important manifestations of the disease are cirrhosis, autoimmune disease, lymphoproliferative conditions such as Mixed Cryoglobulinaemia, and a well-documented association with both low- and high-grade NHLs [154]. HCL accounts for 7.4–37% of NHLs overall [159], the strength of the association varying geographically [160], being particularly high in Italy [161, 162] but absent in some countries, including those of Northern Europe [163], indicating the existence of important environmental cofactors. Populations with high HCV prevalence have a greater propensity to develop HCV-associated NHL [162]. 

It is of particular interest and relevance to this paper that rare instances of sporadic Burkitt's lymphoma arising in connection with chronic HCV infection have been recorded [164–166]. They included a number of primary hepatic BLs [164], a cardiac lymphoma with variant BL translocation and a gingival BL arising in a renal transplant patient with chronic HCV infection [165, 166]. Under-ascertainment of HCV-associated BL is likely, unless there is a high index of diagnostic suspicion. 

### 4.1. Possible Oncogenic Mechanisms of HCV

HCV replicates by way of an RNA-dependent polymerase which lacks a proof-reading function [154]. High rates of genetic variations during replication result in the production of mutant viruses capable of escaping the immune attack and establishing persistent infection. Chronic antigenic stimulation occurs during a lengthy induction period. HCV induces Toll-Like Receptor 4 and consequent enhanced production of Beta-Interferon and Interleukin-6 [167]. HCV directly stimulates B cell expansion, causing a clonal or polyclonal B cell expansion by producing a variety of cytokines and chemokines [167, 168], which may result in mixed cryoglobulinemia, the development of the antiapoptotic *t*(*14;18*) translocation in some patients, and, in a few cases, NHL [162, 168, 169]. 

The virus can greatly enhance mutations of both immunoglobulins and proto-oncogenes by inducing error-prone polymerases and acting on cellular enzymes to enhance production of Nitrous Oxide leading to DNA double-strand breaks, hypermutation of immunoglobulin, proto-oncogene, and tumor suppressor genes, with amplification of the mutated proto-oncogenes [170, 171]. HCV infection inhibits multiple DNA repair processes [172]. Chromosomal abnormalities and polyploidy are frequently found in HCV-infected peripheral blood cells and HCV is thought to inhibit the mitotic checkpoint [173]. The HCV Core and NS3 proteins are responsible for the inhibition of DNA repair, mediated by nitric oxide and reactive oxygen species and both have oncogenic potential, since they can transform certain cell lines [174–176]. The Core Protein can impair cell cycle regulation *in vivo*, affecting the function of human pRb/p105 and other cell growth regulatory proteins, thus uncoupling cell cycle progression from mitotic control and permitting random mutations and rearrangements of the genome [175, 176]. Part of the HCV genome encoding the nonstructural protein NS3 is involved in cell transformation as cells expressing this sequence proliferated rapidly, displayed characteristics associated with malignancy, and were tumorigenic in nude mice [174, 176]. The HCV NS5A protein is also thought to have oncogenic potential, by opposing *TP53 *and acting as a *BCL2* homologue [177, 178]. The HCV protein E2 enhanced the expression of antiapoptotic *BCL2* family proteins and increased the expression of costimulatory molecules CD80, CD86, and CD81, both of which mechanisms are likely to contribute to HCV-associated B cell lymphoproliferative disorders [162]. Thus, HCV chronic infection acts in a number of different ways, resulting in B cell activation and a subset of cells which are more likely to express *BCL2* and to be intrinsically resistant to apoptosis [162, 177–180].

### 4.2. Pathogenesis

The frequency of HCV-associated NHL is much lower than that of HCV infection, suggesting that additional factors are required for lymphomagenesis, which are likely to include cellular interactions with the virus and its products. HCV-associated Cryoglobulinaemia seems to precede the development of both high- and low-grade NHLs [181, 182] and it has been suggested that particular HCV genotypes may be more prone to develop NHLs [169]. 

HCV directly stimulates B cell expansion, causing a clonal or polyclonal B cell expansion [183]. Serum levels of Rheumatoid Factor were found to be increased in patients with a clonal expansion, suggesting that the expanded B-cell clones belong to the Rheumatoid Factor producing B-cell subset [183, 184] and that, in some cases at least, they can evolve into NHL [185].

Up to half of all HCV carriers have mixed cryoglobulinaemia, composed of HCV antigen and antibody. Cryoglobinaemia, and the severity of disease, appears to be linked to the wide range of antibodies produced in HCV infection, consequent, to some extent upon the frequent genetic mutations that the virus produces in the course of the disease. HCV is also associated with monoclonal gammopathies, particularly when infection is due to Genotype 2a/c [186]. Cryoglobulinaemia is associated with the development of the *t*(*14;18*) translocation which consists of the rearrangement and activation of *BCL2*, the antiapoptotic B-cell lymphoma/leukaemia gene and its juxtaposition with the Immunoglobulin heavy chain gene IgH on chromosome 14 [182]. Development of the *t*(*14;18*) translocation, the commonest form of translocation found in lymphomas, is thought to be favoured by chronic antigenic stimulation [182, 187]. This translocation can occur in normal people without malignancy, suggesting that, on its own, it is insufficient to induce a malignant outcome [182]. Chronic antigenic stimulation is considered to be a factor in the clonal evolution of HCV-associated immunocytomas [187]. Both premalignant and malignant lymphoproliferations in an HCV-infected type II Mixed Cryoglobulinemic patient appear to be sequential phases of an antigen-driven pathological process [188]. Effective antiviral treatment leads to the disappearance of the translocation [189, 190] and in some cases, resolution of the lymphoma [162, 191] highlighting the importance of both translocation and virus in the process of lymphomagenesis and the potential reversibility of the process.

EBV coinfection seems to increase the oncogenicity of HCV, at least as regarding its contribution to the incidence of Hepatocellular carcinoma [192]. HCV replication is enhanced in the presence of EBV [193], due to an interaction with EBNA1, thus increasing the effect of antigen-driven oncogenic processes. EBV could also potentiate the effects of HCV's mutator actions because EBV can rescue error-bearing cells from apoptosis [63]. In addition, EBV-infected B cells tend to accumulate more somatic hypermutations, to have more replacement mutations and to occupy a skewed niche within the memory compartment, due to their exclusion from the CD27(+)IgD(+)IgM(+) subset, which protects them from the immune system, since they cannot be distinguished from uninfected cells [194].

## 5. Similarities between Hepatitis C and Arboviruses

HCV is part of the Family Flaviviridae to which those Arborviruses which are Flaviviruses belong. The arboviruses most closely related to HCV are the Flaviviruses Yellow Fever and Dengue. CHIKV is an Alphavirus, belonging to the Togaviridae Family. Flaviviruses are closely related to Alphaviruses, being previously classified as an Alphavirus subgroup, and were only allocated their own family when sufficient differences were noted [153]. It is conceivable that CHIKV, already known to be potentially oncogenic [137, 139] and additionally those arboviruses, closely related to HCV such as Yellow Fever, might deploy oncogenic mechanisms similar to those of HCV because of their shared characteristics, and that some, or all, related Flaviviruses and Alphaviruses could share such potential. 

It has been shown that Arboviruses, as a group, can exhibit persistence and initiate autoimmune disease [119, 126, 127]. CHIKV, as demonstrated during the recent epidemic [121–123], can persist and give rise to chronic infection. Not only autoimmune disease, but also cryoglobulinaemia, has been found to be common in chronic forms of this infection [195]. CHIKV infection, like Dengue, has the ability to induce Haemorrhagic and Shock Syndrome forms of disease [131, 132] which both unleash a huge release of cytokines [132, 196, 197]. Both are thought to be related to crossreactivity with antibodies produced as a result of previous exposure to closely related serotypes of the virus [131–133, 198–201]. Antibody-dependent immune enhancement can also occur during infection, resulting in high levels of replicating virus. Arboviruses have a rapid replication cycle of four hours and, as with HCV, they generate a high rate of genetic variations during viral replication resulting in the production of mutants capable of escaping attack by the immune system. This process is also likely to generate faulty cells requiring either DNA repair or apoptosis, particularly as arboviral RNA polymerases do not have proof-reading ability. If antibody-dependent immune enhancement occurred, it could produce a rapid increase in viral replication and infected cells, unleashing prodigious amounts of cytokines [132], which could exert effects such as those seen in chronic antigenic stimulation. This could challenge the capacity of cellular DNA repair mechanisms at the very least. 

In the Lymphoma Belt setting, chronic EBV infection would provide an expanded pool of B-lymphocytes, thought to be a key factor in lymphomagenesis, because of the enhanced potential for the development of chromosomal abnormalities. This effect would have been amplified still further by the mitogenic effect of holoendemic malaria. CHIKV infection has been shown to be associated with the onset of eBL [12, 22] and an acute arboviral infection could be the reason that the BL cell that is actively rearranging its IgG genes [37]. It is conceivable that acute CHIKV infection in such a setting, particularly if the infection had been preceded by infection with a closely related arbovirus, could initiate a release of cytokines which could have an effect analogous to the antigenic stimulation seen in HCV infection. CHIKV has a very short replication time and also readily produces mutations as its polymerase lacks a proof-reading function. In addition, EBV could cooperate with the arbovirus, by helping error-bearing cells to survive and might also assist the arbovirus to establish persistent infection, as seen *in vitro* [138]. EBV is known to increase the rate of HCV replication [193] but it is unknown whether it exerts a similar effect on arboviruses, though conceivable that this might be the case with those arboviruses closely related to HCV.

Arboviruses readily act as vectors for heterologous genes [134], suggesting the possibility that they could act as vectors within the cell, possibly in conjunction with EBV. Their association with autoimmune disease raises the possibility that they could interact with cellular mechanisms though molecular mimicry, thought to be a factor in autoimmune disease [202].

## 6. Conclusion

A role in lymphomagenesis has been confirmed for HCV [5] and is probable for Hepatitis G [158], suggesting that closely related flaviviruses, such as Yellow fever, and other related groups of arboviruses, could also have lymphomagenic potential. CHIKV is already known to have oncogenic potential [137, 139]. High levels of CHIKV activity were documented around the time when space-time case clusters of eBL were occurring in Malawi [105], and there was a statistically significant association between recent infection with that virus and the onset of eBL [12, 22]. High levels of CHIKV activity were also recorded in NW Cameroon [203] around the time when extremely high rates of eBL were recorded, up to 20/100,000, and spatial clusters were observed [100]. Although no analysis for space-time clustering was performed in Cameroon, it is likely that this was occurring, particularly in one area where the spatial clustering was very pronounced. In addition, the early space-time case clusters recorded in Uganda occurred at a time when epidemic CHIKV [114] and O'nyong-Nyong Virus activity was observed [200, 202]. O'nyong-nyong, like CHIKV, is an Alphavirus and is antigenically extremely closely related to CHIKV [112]. It could appear that not only CHIKV, but possibly both viruses, could be linked with the eBL case clusters seen contemporaneously with their epidemics. It is also possible that CHIKV could have acted as a cofactor for late-stage eBL pathogenesis, in view of the link between recent CHIKV infection and the onset of eBL recorded in Malaŵi [12, 22]. 

As mentioned previously, CHIKV has the potential to be oncogenic since it can transform mouse brain cells [135] and is tumorigenic in nude mice [138]. CHIKV has also recently been shown to give rise to cryoglobulinaemia [195], a lymphoproliferative state analogous to the Mixed Cryoglobulinaemia seen in chronic HCV infection. HCV-associated Mixed Cryoglobulinaemia is thought to be associated with a 35-fold risk of lymphoma development and to evolve into HCV-associated NHL in 8–10% of cases [162]. In eBL lymphomagenesis, oncogenic arboviruses might interact synergistically with EBV, possibly aided by exposure to tumour-promoting, EBV-activating plant extracts [22, 54]. Further research needs to be done to investigate the association between CHIKV, possibly other arboviruses, and eBL. The requisite research is difficult to carry out in the absence of epidemics, which only occur at lengthy intervals, but is long overdue. Such work is likely to elucidate the mechanisms of lymphomagenesis, not only in eBL, but also in sporadic BL. HCV has been shown to be associated with a few cases of sporadic BL and other RNA viruses, apart from HIV, might also contribute to the small number of sporadic BL cases.

**Figure 1:**
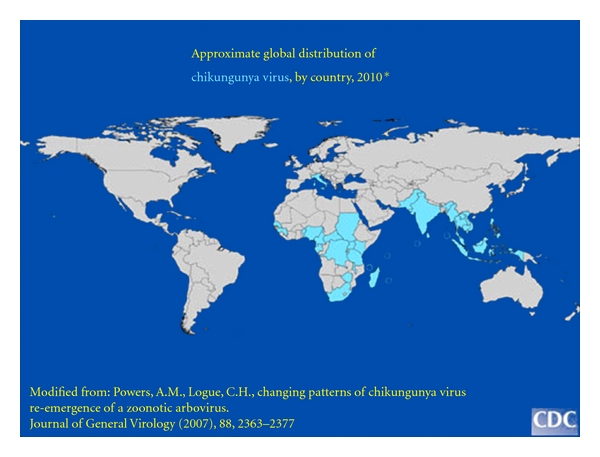


Alphaviruses causing disease similar to CHIKV, are not confined to the Lymphoma Belt, but, as shown in Figure 1, are found in Asia, Australia, the Americas, and Europe. CHIKV vectors are currently extending their range considerably and it would be of interest to see how much an updated map of the geographic distribution of eBL differed from the Lymphoma Belt of Africa as originally defined by Burkitt in the 1950s [204]. A CHIKV outbreak recently occurred in Northern Italy [205], where the vector, *Aedes albopictus*, is now endemic. Local transmission in Italy and new areas invaded by the virus, may offer the dubious advantage of providing arboviral research material where pre-existing expertise and research facilities are readily available. Our knowledge about chronic CHIKV disease has already advanced due to the recent epidemic in the Indian Ocean and India [195], and it is to be hoped that progress will also be made with the assessment of the oncogenic potential of this hitherto underestimated virus. 

Global warming and other factors contingent on the emergence of infectious organisms, and viruses in particular, will almost certainly contribute to an increased disease burden [206] in future, due not only to acute infection, but also the more challenging, often initially inapparent, sequelae of chronic infections. It is likely that more infectious agents, particularly viruses, including some yet to be identified, will be implicated in lymphomagenesis and oncogenesis and their study will continue to illuminate oncogenic processes, aided by advances in molecular biology and improved diagnostic methods.

## Figures and Tables

**Table 1:** Reports of Case clustering in endemic Burkitt's Lymphoma.

Location	Dates	Space only	Space and time	Author
West Nile,	1961–65		+	Williams et al. [97, 98], Pike 1972,
Uganda	1972-73			Siemiatycki et al. [104]
Mengo District and Bwamba County, Uganda	1966–68		+	Morrow et al. [103, 106], 1974
Aliba, Uganda	1962-63		+	Pike et al. [101]
Malawi	1987–90		+	van den Bosch [22, 109]
West Kenya	1999–04	+	Not tested	Rainey et al. [99]
Cameroon	2003–2006	+	Not tested	Wright et al. [100]

**Table 2:** Arboviruses and Malaria—a comparison.

Characteristic	Arboviruses	Malaria
Epidemiology in Lymphoma Belt	Endemic and occasionally epidemic	Holoendemic + 3 types less intense
Age acquisition immunity	Mimics age distribution of eBL	By age of 5 yrs if holoendemic
Altitude Barrier	5000 ft at Equator, 3000 ft in Zambia—same as BL	Up to 8,000 ft at Equator
Geographic Distribution	Dependent on vectors—usually mosquitoes	Dependent on anopheline mosquitoes
Replication Temperature Requirements	Yellow fever stops <15.5–18°C	Malaria stops <20°C
	Same as BL	(PF > 18°C, PV > 17°C, PM > 16°C)
Effect malaria suppression	None	Reduced
Effect malaria eradication	Eradicated	Eradicated

**Table 3:** Characteristic arboviral signs and symptoms seen in eBL patients immediately preceding development of lymphoma.

Sign or symptom	Total number (%)	Time before BL in days (range)	CHIK IgG/M+ On admission	CHIK IgG/M+ after 14/7
Rash	9 (8)	8 (2–14)	0	5
Sore eyes	16 (18)	19 (7–28)	1	10
Joint pains	32 (37)	14 (2–28)	9	16
Mouth ulcers	14 (16)	13 (3–21)	2	10
Fever	27 (31)	16 (1–56)	10	19
Bleeding	14 (16)	19 (14–28)	3	6

**Table 4:** Classification of arboviruses.

Family	Genus	Disease	Vector
Flaviviridae	Flavivirus Formerly Casal's Gp B	Yellow Fever Dengue Japanese Encephalitis Saint-Louis Encephalitis West Nile Fever Tick-borne Encephalitis	Aedes Mosquitoes Aedes Mosquitoes Culicine Mosquitoes Culicine Mosquitoes Culicine Mosquitoes Ticks
Togaviridae	Alphaviruses Formerly Casal's Gp. A	Chikungunya, O'Nyong-Nyong, Sindbis Ross River Fever, Barmah Forest	Aedes and Culicine Mosquitoes
		Mayaro	Culicine Mosquitoes
		Equine Encephalitis	
Bunyaviridae	Bunyavirus Nairovirus Phlebovirus	Bunyamwera Virus Crimea-Congo Haemorrhagic Fever Rift Valley Fever Sand-fly Fever	Aedes Mosquitoes Ticks Mosquitoes and Ticks Sandflies
